# Emergency and critical care professionals’ opinion on escape room as a health sciences evaluation game: A cross-sectional descriptive study

**DOI:** 10.1097/MD.0000000000029432

**Published:** 2022-06-24

**Authors:** Jose L. Gómez-Urquiza, Iván Requena-Palomares, Esther Gorjón-Peramato, Juan Gómez-Salgado, Guillermo A. Cañadas-De la Fuente, Luis Albendín-García

**Affiliations:** a Nursing Department, Faculty of Health Sciences, University of Granada, Granada, Spain; b Medical Emergency Service, Catalan Health Service (CatSalut), Barcelona, Spain; c Medical Emergency Service, Madrid Health Service (SUMMA112), Madrid, Spain; d Department of Sociology, Social Work and Public Health. Faculty of Labour Sciences, University of Huelva, Huelva, Spain; e Safety and Health Posgraduate Programme. University Espíritu Santo, Guayaquil, Ecuador; f La Chana Health Center, Andalusian Health Service, Junta de Andalucia, Granada, Spain.

**Keywords:** education, emergency nurse, emergency physician, escape room, game-based learning, health sciences

## Abstract

New teaching and evaluation methods are growing in health sciences. The escape room is a game that is showing benefits for assessing knowledge and important competencies in healthcare professionals. The aim of this study is to analyse the opinion of emergency and critical care professionals on the use of escape rooms as an evaluation game.

A quantitative, descriptive, cross-sectional study was conducted using an ad-hoc questionnaire with a Likert-type scale. The study included emergency and critical care professionals who participated in the escape room “The Frustrated Emergency and Critical Care Professional,” that took place during an emergency and critical care national congress. Data collection was carried out in June 2019.

The sample was composed of n = 50 emergency and critical care professionals, 52% of whom were physicians and 48% were nurses. Professionals believe that this is a good teaching game for evaluation and useful for strengthen knowledge (4.7 points), as well as to improve teamwork and the ability to work under pressure (4.9).

The escape room is a useful evaluation game in the context of emergency and critical care units that also allows training the teamwork and working under pressure competencies.

## 1 Introduction

University teaching is undergoing changes aimed at gaining more effective learning, with more attractive and motivating classes for students.^[1]^ Against the teaching through a master classes approach, methods that seek to improve learning through greater involvement of students in the process^[2]^ are being applied. Among others, we find gamification, by including game elements or dynamics in processes or objectives, that are not related to them, to make them more attractive^[3]^; flipped learning, also known as inverted learning, in which part of the agenda is worked at home before going to class and the class is used as a more dynamic means; or game-based learning, which uses games as a tool for motivation and help in the learning process.

The interest in games is growing because, through motivation, they capture interest and promote active involvement and student thinking.^[4]^ Some examples of teaching games in health sciences report on the use of a television quiz show to review^[5]^; and others use *serious online games* in decision-making and clinical reasoning.^[6]^

The escape room is a game that is gaining a lot of prominence today for its positive results on satisfaction, motivation, and usefulness for learning, in addition to promoting skills such as leadership, the ability to work under pressure, and teamwork.^[7]^ This game consists of “locking up” students or professionals to be evaluated in groups of 5 in a classroom. They must solve different tests, riddles and puzzles related to the knowledge and techniques of a certain subject or specialty.^[7]^ This game has been tested with radiology and surgery residents,^[8,9]^ to work on issues related to patient safety,^[10]^ and with pharmacy and nursing students.^[7,11]^ However, as far as we know, there is no literature on their use in emergency and critical care professionals. Therefore, and for the benefits of game-based learning and escape rooms mentioned above, this study is proposed.

The objective was to analyse the opinion and satisfaction with the escape room as a knowledge assessment game in emergency and critical care professionals.

## 2 Methods

### 2.1 Study design and sample selection

A quantitative, descriptive, cross-sectional study was carried out. The selection of the sample was intentional and was formed by emergency and critical care professionals who participated in the escape room carried out at a national emergency and critical care congress. Participants in the game were included without exclusion criteria by profession, age, or sex.

### 2.2 Rules and development of the escape room

The game was titled “The Frustrated Emergency and Critical Care Professional” and participants had to find a law that had not yet been passed which laid down the foundations for the creation of the emergency and critical care specialty.

The groups were made up of 5 people each, and the time to solve the game and escape was 30 minutes, providing a clue after 10 minutes, and another one after 20 minutes.

**Figure 1:**
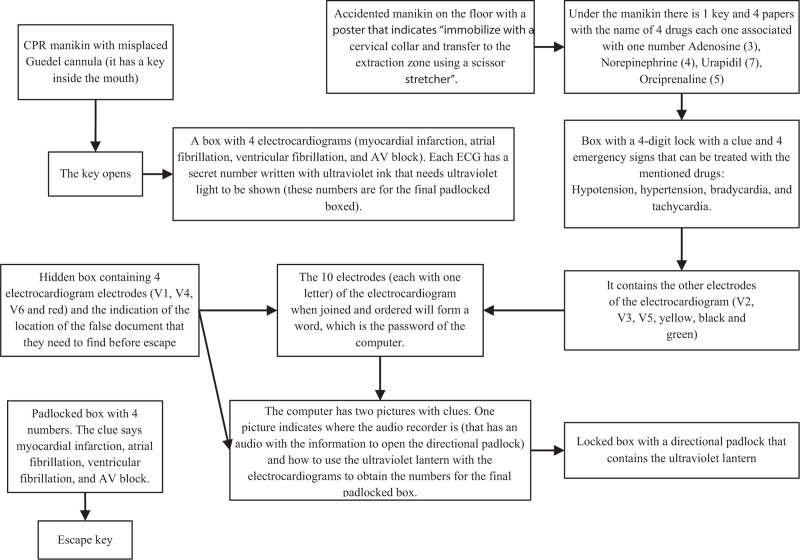
Escape room scheme.

The emergency and critical care knowledge included in the game was formed by the transfer of the critical patient, taking, reading and diagnosis of electrocardiograms, drugs for urgent pathologies, and immobilisation devices in out-of-hospital emergencies. A general scheme of the game is shown in Figure 1.

### 2.3 Variables and data collection procedure

A printed self-administered ad-hoc questionnaire was used for data collection and delivered to the participants in the escape room.

Data collection was carried out in June 2019 with professionals from a national emergency and critical care congress. The sociodemographic variables sex and age were collected, as well as the labour variable profession and, as a result, and based on previous questionnaires from similar studies,^[7,11]^ the following questions were assessed using a Likert-type scale of 5 points (1 = strongly disagree, and 5 = strongly agree):

(1) It is a useful game for learning in emergency and critical care units.
(2) Allows to evaluate theoretical-practical knowledge on emergencies and critical care.
(3) It helps strengthen knowledge in emergencies and critical care.
(4) I believe that it can increase students’ motivation.
(5) It is a good game for developing teamwork.
(6) It is a good game to train reaction and thinking under pressure.
(7) I am satisfied with the game.
(8) I consider developing an escape room as a teaching method in the future.

### 2.4 Data analysis

Descriptive analyses of continuous quantitative variables (mean and standard deviation) and frequency analysis for categorical variables were performed with the Statistical Package for the Social Sciences 22 software (IBM, Armonk, NY). A *t*-student test for independent samples was used for comparing the scores between the profession, sex and teaching experience variables.

### 2.5 Ethical aspects

All methods were performed in accordance with the Helsinki Declaration of Ethical Principles in Human Research Guidelines (Fortaleza, 2013) and the Spanish regulations for biomedical research. The University of Granada and the Faculty of Health Sciences gave their consent for doing the nursing escape room. Also, this study was approved by the Ethics Committee of the University of Granada. The participation in the escape room and in the questionnaire was voluntary and anonymous. Appropriate written informed consents were obtained from the participants.

## 3 Results

The sample consisted of n = 50 emergency and critical care professionals (90% response rate), 52% of whom were physicians and 48%, nurses. The mean age of the sample was 37.48 years (standard deviation = 8.2), 66% were females and 24% were males. Regarding the teaching work of these professionals, 84% said they had at some point taught health sciences students or health professionals and had held this role in the last year.

Regarding the questions about the developed game, the ones that obtained the highest mean were the items “Helps strengthen knowledge in emergencies and critical care’,” “It is a good game to train reaction and thinking under pressure” and “I am satisfied with the game.” The mean scores for the items are shown in Table 1.

**Table 1:** Mean scores of the questionnaire.

Items	Mean score	Standard deviation
1. It is a useful game for learning in urgent care and emergency departments	4.76	1.1
2. Allows to evaluate theoretical-practical knowledge on urgent care and emergencies	4.74	0.59
3. Helps strengthen knowledge in urgent care and emergencies	4.9	0.56
4. I believe it can increase students’ motivation	4.6	0.6
5. It is a good game for developing teamwork	4.86	0.58
6. It is a good game to train reaction and thinking under pressure	4.9	0.60
7. I am satisfied with the game	4.9	0.62
8. I consider developing an escape room as a teaching method in the future	4.66	0.58

1 = strongly disagree; 2 = disagree; 3 = not agree nor disagree; 4 = agree; 5 = strongly agree.

When comparing the answers between physicians and nurses, only the item “*It is a useful game for learning in emergency and critical care units*” showed statistically significant higher score in nurses (mean score 4.9) than in physicians (mean score 4.01) with a *P*-value <.05. No statistically significant differences were found depending on sex or whether the professional had teaching experience or not.

## 4 Discussion

The objective of the study was to analyse the opinion of emergency and critical care professionals on the use of escapism or escape rooms as a teaching game for assessing the contents of this specialty. Regarding the results, it can be said that emergency health professionals consider the escape room a useful game for the assessment of their specialty, in addition to strengthening knowledge and developing skills such as teamwork and working under pressure. Similar results have been found in medical residents, nursing students and undergraduate pharmacy students.^[7,8,12]^ Although this method is positive for varying the types of evaluation and it will not benefit everyone as there are different learning profiles depending on each student.

In other games found in the literature, such as online simulation websites or quiz games, students’ experience is also positive.^[13]^ However, it is worth noting that the escape room is a more dynamic activity in which students have complete access to the whole classroom and, as time is running, they move freely trying to solve and overcome the game.^[7]^

As we have seen, the escape room also allows training competences such as teamwork and working under pressure, which are basic competencies in health care professionals.^[14]^ Such skills are usually worked in clinical simulation environments; however, the cost of a simulation classroom is more expensive.^[15]^ while an escape room only needs investment in some items such as padlocks, lockable boxes, or consumables.

One aspect that can influence the development of escape rooms by teachers in courses on urgent care and emergencies is the time investment this type of dynamics imply against master classes or classic examinations methods, to which teachers are usually more accustomed. This variable, the cost of time and the future efficiency in learning, can be a barrier for some teachers since the escape room may mean a greater number of hours in the classroom because each group can only be made up of 5 people, to which we would have to add up the time to develop it and to plan it at home. This can be considered a negative point when compared to other games in which all students can perform at once.

This study has some limitations. The study sample is not very extensive, because the time for group participation in the game was very limited and no more groups were able to participate in it. With regard to the questionnaire, a self-administered one was used, although it was based on similar literature on the subject.^[7]^ Finally, this study only shows the reaction of the participants and thus the results must be considered taking this into account.

Future lines of research could analyse the effectiveness of this game to assess knowledge as compared to the usual written exams. Studies could also be conducted to assess teacher concordance as evaluators in identifying group leaders and assessing teamwork and under pressure work from each group.

## 5 Conclusions

Emergency and critical care professionals who participated in the escape room believe that this is a very useful game for the evaluation of knowledge and techniques, in addition to promoting other skills such as teamwork, working under pressure, and leadership. This game could be implemented in undergraduate and postgraduate training in emergencies and critical care so that teachers can evaluate content differently from the classic exam, as well as assess basic competencies.

The “Escape Room” is a useful teaching game that stimulates learning, is fun to participate and motivates teamwork among other competencies, while implies the use of several different clinical techniques and proceedings on many diverse scenarios. Some of the most valued competencies by nurses were emotional and psychological.

## Acknowledgments

The authors want to thank SEMES for the opportunity of developing and executing the escape room during their congress.

## Author contributions

**Conceived the ideas:** Jose L. Gómez-Urquiza, Iván Requena-Palomares, and Esther Gorjón-Peramato.

**Collected the data:** Jose L. Gómez-Urquiza, Iván Requena-Palomares, Esther Gorjón-Peramato, and Guillermo A. Cañadas-De la Fuente.

**Analysed the data:** Jose L. Gómez-Urquiza, Iván Requena-Palomares, Esther Gorjón-Peramato, Juan Gómez-Salgado, Guillermo A. Cañadas-De la Fuente, and Luis Albendín-García.

**Led the writing:** Jose L. Gómez-Urquiza, Iván Requena-Palomares, Esther Gorjón-Peramato, Juan Gómez-Salgado, Guillermo A. Cañadas-De la Fuente, and Luis Albendín-García.

Final approval was obtained from all authors.

## References

[1] Biggs J Tang C Kirby J. Teaching for Quality Learning at University. 4th ed New York: McGrawHill, ed; 2011.

[2] Chi MTH Wylie R. The ICAP framework: linking cognitive engagement to active learning outcomes. Educ Psychol 2014;49:219–43.

[3] Day-Black C Merrill EB Konzelman L Williams TT Hart N. Gamification: an innovative teaching-learning strategy for the digital nursing students in a community health nursing course. ABNF J 2015;26:90–4. 26665503

[4] Garris R Ahlers R Driskell JE. Games, motivation, and learning: a research and practice model. Simul Gaming 2002;33:441–67.

[5] Boctor L. Active-learning strategies: the use of a game to reinforce learning in nursing education. A case study. Nurse Educ Pract 2013;13:96–100. 22910398 10.1016/j.nepr.2012.07.010

[6] Johnsen HM Fossum M Vivekananda-Schmidt P Fruhling A Slettebø Å. Teaching clinical reasoning and decision-making skills to nursing students: design, development, and usability evaluation of a serious game. Int J Med Inform 2016;94:39–48. 27573310 10.1016/j.ijmedinf.2016.06.014

[7] Gómez-Urquiza JL Gómez-Salgado J Albendín-García L Correa-Rodríguez M González-Jiménez E Cañadas-De la Fuente GA. The impact on nursing students’ opinions and motivation of using a “Nursing Escape Room” as a teaching game: a descriptive study. Nurse Educ Today 2019;72:73–6. 30453202 10.1016/j.nedt.2018.10.018

[8] Jambhekar K Pahls RP Deloney LA. Benefits of an escape room as a novel educational activity for radiology residents. Acad Radiol 2020;27:276–83. 31160173 10.1016/j.acra.2019.04.021

[9] Kinio AE Dufresne L Brandys T Jetty P. Break out of the classroom: the use of escape rooms as an alternative teaching strategy in surgical education. J Surg Educ 2019;76:134–9. 30126728 10.1016/j.jsurg.2018.06.030

[10] Zhang XC Diemer G Lee H Jaffe R Papanagnou D. Finding the ‘QR’ to patient safety: applying gamification to incorporate patient safety priorities through a simulated ‘escape room’ experience. Cureus 2019;11:e4014. 31007972 10.7759/cureus.4014 PMC6453616

[11] Clauson A Hahn L Frame T. An innovative escape room activity to assess student readiness for advanced pharmacy practice experiences (APPEs). Curr Pharm Teach Learn 2019;11:723–8. 31227096 10.1016/j.cptl.2019.03.011

[12] Eukel HN Frenzel JE Cernusca D. Educational gaming for pharmacy students – design and evaluation of a diabetes-themed escape room. Am J Pharm Educ 2017;81:6265. 29109566 10.5688/ajpe8176265 PMC5663657

[13] Maheu-Cadotte M-A Cossette S Dubé V. Effectiveness of serious games and impact of design elements on engagement and educational outcomes in healthcare professionals and students: a systematic review and meta-analysis protocol. BMJ Open 2018;8:e019871. 10.1136/bmjopen-2017-019871 PMC5857654 29549206

[14] Babiker A El Husseini M Al Nemri A. Health care professional development: working as a team to improve patient care. Sudan J Paediatr 2014;14:09–16. PMC4949805 27493399

[15] Al-Elq AH. Simulation-based medical teaching and learning. J Family Community Med 2010;17:35–40. 22022669 10.4103/1319-1683.68787 PMC3195067

